# Cytokine-Like Factor 1, an Essential Facilitator of Cardiotrophin-Like Cytokine:Ciliary Neurotrophic Factor Receptor α Signaling and sorLA-Mediated Turnover

**DOI:** 10.1128/MCB.00917-15

**Published:** 2016-04-01

**Authors:** Jakob Vejby Larsen, Anders Mejer Kristensen, Lone Tjener Pallesen, Johannes Bauer, Christian Bjerggaard Vægter, Morten Schallburg Nielsen, Peder Madsen, Claus Munck Petersen

**Affiliations:** a The MIND Center, Department of Biomedicine, Aarhus University, Aarhus, Denmark; b The MIND Center, Department of Molecular Biology and Genetics, Aarhus University, Aarhus, Denmark

## Abstract

Cardiotrophin-like cytokine:cytokine-like factor-1 (CLC:CLF-1) is a heterodimeric neurotropic cytokine that plays a crucial role during neuronal development. Mice lacking CLC:CLF-1 die soon after birth due to a suckling defect and show reduced numbers of motor neurons. Humans carrying mutations in CLC:CLF-1 develop similar disorders, known as Sohar-Crisponi or cold-induced sweating syndrome, and have a high risk of early death. It is well known that CLC binds the ciliary neurotrophic factor receptor α (CNTFRα) and is a prerequisite for signaling through the gp130/leukemia inhibitory factor receptor β (LIFRβ) heterodimer, whereas CLF-1 serves to promote the cellular release of CLC. However, the precise role of CLF-1 is unclear. Here, we report that CLF-1, based on its binding site for CLC and on two additional and independent sites for CNTFRα and sorLA, is a key player in CLC and CNTFRα signaling and turnover. The site for CNTFRα enables CLF-1 to promote CLC:CNTFRα complex formation and signaling. The second site establishes a link between the endocytic receptor sorLA and the tripartite CLC:CLF-1:CNTFRα complex and allows sorLA to downregulate the CNTFRα pool in stimulated cells. Finally, sorLA may bind and concentrate the tripartite soluble CLC:CLF-1:CNTFRα complex on cell membranes and thus facilitate its signaling through gp130/LIFRβ.

## INTRODUCTION

The heterodimeric cytokine cardiotrophin-like cytokine:cytokine-like factor-1 (CLC:CLF-1) is a member of the human interleukin-6 (IL-6) family of cytokines, which also includes ciliary neurotrophic factor (CNTF), cardiotrophin, leukemia inhibitory factor (LIF), oncostatin M, IL-6, IL-11, and IL-31 (1). The family members are structurally related, and following binding to separate but mutually related (cytokine type 1) membrane receptors, they recruit the transmembrane glycoprotein 130 (gp130) and, in most cases, the LIF receptor β (LIFRβ), for signal transduction (1, 2). CLC:CLF-1 is composed of two independently expressed proteins, CLC (alias, novel neurotrophin-1 or B cell-stimulating factor-3) and CLF-1 (alias, cytokine receptor-like factor 1) (3–5). Mature CLC consists of 198 amino acids with ∼28% sequence identity to CNTF and forms a four-helical bundle. It is synthesized with a signal peptide and is expressed in the biosynthetic pathway of a variety of cell types but is poorly secreted. Like CNTF, CLC binds to the CNTF receptor α (CNTFRα), either the glycosylphosphatidylinositol (GPI)-anchored or the soluble form, and elicits signaling via interaction with gp130/LIFRβ heterodimers and subsequent activation of the Janus kinase (JAK) and the signal transducer and activator of transcription-3 (STAT3) pathway (6). Human CLF-1 is a glycosylated soluble 385-amino-acid protein with significant similarities to cytokine type 1 receptors like the IL-6 receptor α (IL-6Rα). On its own, CLF-1 does not appear to induce signaling, but several lines of evidence have established that it plays a crucial role in the cellular and physiological functions of CLC by binding CLC in a stable complex (4, 7, 8). When coexpressed, CLF-1 and CLC interact in the biosynthetic pathway, and CLF-1 serves to facilitate rapid transport and secretion of the complex (4). Alternatively, CLF-1 may target free CLC released from cells or bound to plasma membranes via the CNTFRα. In any case, the CLC:CLF-1 heterodimer binds CNTFRα, and compared to CLC:CNTFRα, the tripartite complex significantly enhances gp130/LIFRβ activation and STAT3 phosphorylation in cells (4, 6). Thus, CLF-1 promotes not only the cellular secretion of CLC but also its induction of transmembrane signaling.

The close functional connection between CNTFRα, CLC, and CLF-1 is strongly reflected by findings *in vivo* showing that mice deficient in any of the three proteins display the same phenotype (7, 9, 10). The mice all fail to suckle, die soon after birth, and show reduced numbers of motor neurons in the facial nucleus and lumbar ventral horns (7, 9–11). Moreover, human patients carrying mutations (single homozygotes or compound heterozygotes) in CLC or CLF-1 (8, 12, 13) develop a series of similar manifestations known as Sohar-Crisponi or cold-induced sweating syndrome (CISS) (14–16), which includes feeding difficulties and risk of early death. However, despite the apparent overlap between CLC and CLF-1 functions, the precise role of CLF-1 in the tripartite complex with CNTFRα and CLC is insufficiently understood, and recent findings even suggest that CLF-1 may have separate functions and alternative partners (17, 18). Thus, CLF-1 has been reported to complex with other cytokine components, and it is still an open question if it (on its own) may target hitherto unidentified receptors (19).

In humans CNTFRα is the common primary receptor for CNTF and CLC:CLF-1, and we have previously reported that both ligands also bind the membrane receptor sortilin, which mediates their uptake and appears to promote their induction of gp130/LIFRβ and JAK/STAT3 signaling in transfected cells (20). Preliminary unpublished findings further indicate that CLC:CLF-1, but not CNTF, engages the sortilin-related multiligand receptor sorLA, which is widely expressed both inside and outside the nervous system.

Along with sorCS1 to sorCS3, sortilin and sorLA constitute the Vps10p-domain (Vps10p-D) family of type 1 membrane receptors (21). The family hallmark is the N-terminal Vps10p-D comprising a unique neuropeptide and protein-binding 10-bladed β-propeller supported by two minor domains (22). The Vps10p-D constitutes the entire luminal part of sortilin, whereas sorLA contains a series of other structural elements, notably, a small β-propeller domain with an associated epidermal growth factor class B-like motif, followed by a cluster of 11 low-density lipoprotein receptor (LDLR) class A repeats (23, 24). Similar structural elements are typical for members of the LDLR family, and accordingly ligands like receptor-associated protein (RAP), platelet-derived growth factor bb, lipoprotein lipase, apolipoprotein E, and proteins of the plasminogen activator system that target members of this family also target sorLA (25, 26). The cytoplasmic tail of sorLA is short but contains several functional motifs for binding of cytoplasmic adaptor proteins (e.g., AP-1 and -2, GGA1 to GGA3, and elements of the retromer complex) involved in receptor trafficking (27–29). Accordingly, sorLA conveys rapid endocytosis and cellular uptake of ligands bound at the surface membrane as well as transport between *trans*-Golgi compartments and endosomes of newly synthesized protein bound in the biosynthetic pathway (27, 30). Interestingly, sorLA targets not only soluble ligands but also membrane-associated and transmembrane proteins. It is well documented that complex formation with the amyloid precursor protein (APP) affects APP processing and the generation of Aβ-amyloid peptide, and recent findings provide evidence that sorLA's interaction with glia cell line-derived neurotrophic factor (GDNF) and its primary receptor, GDNF family receptor α, may impact receptor tyrosine kinase RET-mediated signaling *in vivo* (31, 32). It appears that sorLA is a multiligand receptor displaying a diversity of cellular functions which, depending on the local expression pattern of ligands and receptors, may involve tissues both inside and outside the nervous system.

In the present report we have examined interactions between the elements of the tripartite complex CLC:CLF-1:CNTFRα, the capacity of the individual subunits for binding to sorLA, and the functional implications of these interactions in neuronal and nonneuronal cells. Our findings reveal that CLF-1 promotes CLC's association with CNTFRα, that CLF-1, in complex or on its own, exhibits a high-affinity site for binding to sorLA, and that its binding to sorLA has a profound effect on the turnover and signaling of CLC:CNTFRα.

## MATERIALS AND METHODS

### Reagents.

Recombinant soluble CNTFRα (sCNTFRα), CLC, CLC:CLF-1, covalently linked CLC-sCNTFRα fusion protein (all human reagents), and goat anti-CNTFRα were purchased from R&D Systems. Neurotensin (NT), leupeptin, pepstatin A, and mouse anti-β-actin were from Sigma. Rabbit anti-CLF-1 and rabbit anti-CLC were purchased from Abcam. The mouse anti-STAT3, rabbit anti-phosphor-STAT3 (Tyr705) (pSTAT3), and horseradish peroxidase (HRP)-linked anti-rabbit and anti-mouse antibodies were from Cell Signaling Technology. Mouse anti-His and anti-Myc tag antibodies were purchased from Genscript, and mouse anti-LAMP-1 (H4A3) was from DSHP. The murine monoclonal anti-sorLA was generated against the cytoplasmic domain of sorLA (30), and the rabbit polyclonal anti-sorLA was generated against the Vps10p-D of sorLA (27). Secondary antibodies conjugated with Alexa Fluor dyes (488 or 568) were all purchased from Invitrogen. PCR and restriction products were purified using Nucleobond from Macherey-Nagel.

### cDNA construct, protein expression, and purification.

Human sorLA glutathione *S*-transferase (GST)–propeptide and RAP were expressed in Escherichia coli and purified as previously described (26, 33). Generated human cDNA constructs encoding soluble sorLA and sorLA Vps10p-D (26) were inserted into pcDNA3.1 expression vectors and expressed in eukaryotic Chinese hamster ovary (CHO) cells as described previously (26). For generation of CNTFRα-Myc, a Myc–tag was inserted between the signal peptide and the gene coding for the mature human CNTFRα protein. The cDNA fragment was inserted in the pcDNA3.1/hyg(−) expression vector and synthesized by MWG Biotech. Human CLF-1 cDNA was purchased from ImaGenes. CLF-1-coding cDNA was amplified by PCR, and a 6×His tag was added at the C terminus using the forward primer 5′-ACA ACA TCT AGA ATG GCC GCC GGC CGC CGG GGC CCC-3′ and the reverse primer 5′-CGC TCT CCA GGA CGG TCT GTA GTG GTA GTG GTA GTG ATT GAG CTC CAC-3′. The PCR-amplified CLF-1 was cleaved with XbaI and XhoI (New England BioLabs) and ligated into pcDNA3.1/zeo(−). For CLF-1–His expression and purification, HEK293 cells transfected with CLF-1–His were grown in Dulbecco modified Eagle's medium (DMEM) (Lonza) until cells were confluent. Afterwards, cells were changed to CCM5 serum free medium (HyClone; Thermo Scientific) for approximately 5 days (until cells started to fall off). The medium was harvested and centrifuged for 30 min at 4,000 × *g* at 4°C. The supernatant was collected and preserved in 0.5% NaN_3_. The spun medium was prepared for purification by the addition of 50 mM Na_2_HPO_4_, 0.3 M NaCl, 10 nM imidazole, and 0.02% Triton X-100 and adjusted to pH 7. The purification was done overnight on a Talon His tag purification resin (Clontech)-prepared XK column (Amersham Biosciences), with a total column volume of 13 ml at 4°C. Before purification from medium, the column was washed in 50 mM Na_2_HPO_4_ and 300 mM NaCl, pH 7.0. The column was then eluted in 50 mM sodium acetate (NaOAc) and 300 mM NaCl, pH 5.0, and collected in 1-ml fractions, where 3 M Tris-HCl, pH 8.8, was added to raise the pH. The concentration of fractions containing CLF-1–His was determined by absorption at 280 nm, and fractions were concentrated on a Centriprep YM-10 instrument (Millipore) to a concentration of 1 mg/ml.

### Cell lines and transfection.

Human embryonic kidney (HEK293) cells were cultured in DMEM supplemented with 10% fetal bovine serum (FBS), 100 U/ml penicillin, and 100 μg/ml streptomycin. HEK293 cells were transfected with pcDNA3.1/zeo(−) and/or pcDNA3.1/hyg(−) using FuGENE 6 (Roche), and stably selected clones were selected in medium containing 150 μg/ml zeocin and/or 500 μg/ml hygromycin B. SH-SY5Y cells were cultured in DMEM–F-12 medium supplemented with 10% FBS, 100 U/ml penicillin, and 100 μg/ml streptomycin and transfected with pcDNA3.1/zeo(−) using FuGENE 6 (Roche). Stably selected clones were selected in medium containing 300 μg/ml zeocin. Murine bone marrow-derived pro-B cells (Ba/F3) containing gp130 and LIFRβ (34), were grown in DMEM supplemented with 10% FBS, 100 U/ml penicillin, 100 μg/ml streptomycin, and 10 ng/ml LIF. The cells were transfected with sorLA by electroporation as described previously (20). Stably selected clones were selected in medium containing 150 μg/ml zeocin and 10 ng/ml LIF.

### Immunocytochemistry and quantitative analysis.

Untransfected and sorLA transfected HEK293 cells were cultured on cover slides and incubated with 10 nM CLC, CLF-1, or CLC:CLF-1 for 25 min at 37°C. The cells were then washed in phosphate-buffered saline (PBS), pH 7.4, fixed in 4% paraformaldehyde (PFA), pH 7, and permeabilized with 0.5% saponin (Sigma). The cells were then incubated with rabbit anti-CLC and/or mouse anti-His and/or mouse anti-sorLA and stained with the matching Alexa Fluor-conjugated secondary antibodies. In some experiments cells were incubated with 0.5 μg/ml bisbenzimide (Hoechst; Sigma) for nucleus staining. All stainings were analyzed by confocal microscopy (LSM710 or LSM780 instrument; Carl Zeiss).

Murine wild-type (wt) and sorLA knockout (KO) (32) astrocytes and hippocampal neurons (all procedures involving animal objects were performed in compliance with Danish and European regulations, and breeding was approved by The Danish Animal Experiments Inspectorate [permit number 2012-15-2935-00007]) were cultured on cover slides and incubated with or without 40 nM CLC:CLF-1 (containing a His tag) for 25 min at 37°C and then washed in PBS, fixed in 4% PFA, permeabilized with 0.5% saponin or Triton X-100, and blocked with FBS. The cells were then incubated with mouse anti-His and rabbit anti-sorLA and stained with appropriate conjugated antibodies.

Untransfected and sorLA transfected SH-SY5Y cells were cultured on cover slides and incubated with or without 10 nM CLC or CLC:CLF-1 (containing a His tag) for 25 min at 37°C. Cells were then washed in PBS, fixed in 4% PFA, permeabilized with 0.5% saponin, and labeled with mouse anti-His and/or goat anti-CNTFRα and/or mouse anti-sorLA, followed by staining with the matching secondary antibodies.

HEK293 cells stably transfected with CNTFRα-Myc or sorLA and CNTFRα-Myc in combination were labeled with mouse anti-Myc for 10 min at 37°C, washed, and then incubated for 0 or 15 min at 37°C in medium with or without 10 nM CLC or CLC:CLF-1. Subsequently, the cells were washed, fixed, and permeabilized, followed by staining using the appropriate secondary antibody.

Quantitative analysis of SH-SY5Y cells was performed on an Olympus ScanR imaging station as described in Klinger et al. (30). In short, all images were acquired with a 40× dry objective, a triple band filter for Hoechst, Alexa Fluor 488, and Alexa Fluor 568, and a Hamamatsu (C8484-05G) camera. The analysis was done on *z*-stack (3-stack) projected images that were background subtracted. An edge detection algorithm was used for segmentation of nuclei and CNTFRα-stained vesicles. Objects were independently selected using only the criteria that they should be able to be encircled in a closed circle (edge) of 3 μm in diameter. The enclosed circles were counted as CNTFRα vesicles. The mean number of CNTFRα vesicles was determined by calculating the ratio of vesicles to nuclei.

### SPR.

Surface plasmon resonance (SPR) analysis was performed on a Biacore 3000 instrument (Biacore, Sweden) equipped with CM5 sensor chips activated as described previously (35). The entire luminal part of sorLA (soluble sorLA), the sorLA Vps10p-D, and sCNTFRα were immobilized to densities of 66 to 86 fmol/mm^2^, and samples for binding (40 μl) were injected at 5 μl/min at 25°C in 10 mM HEPES, 150 mM NaCl, 1.5 mM CaCl_2_, 1 mM EGTA, and 0.005% Tween 20, pH 7.4. Binding was expressed as the difference between the response obtained from the flow cell with immobilized receptor and the response obtained when an activated but uncoupled chip was used. The overall *K_d_* (dissociation constant) values were determined by BIAevaluation, version 4.1, software using a Langmuir 1:1 binding model and simultaneous fitting to all curves in the concentration range considered (global fitting).

### Cross-linking and coimmunoprecipitation.

Transfected HEK293 cells were washed with PBS, followed by cross-linking in 45 min with 2 nM dithiobis(succinimidyl propionate) (DSP). The reaction was stopped at 100 mM Tris, pH 7.5, and cells were washed twice with PBS. Cells were subsequently lysed at 4°C in 1% Triton X-100 lysis buffer (20 mM Tris-HCl, 10 mM EDTA, pH 8.0) supplemented with proteinase inhibitor cocktail (Complete Mini; Roche), and precipitations were performed using rabbit anti-sorLA and mouse anti-Myc antibodies. The samples were subjected to immunoblot analysis using mouse anti-sorLA and goat anti-CNTFRα antibodies.

### Downregulation of CNTFRα.

HEK293 cells stably transfected with CNTFRα-Myc or sorLA and CNTFRα-Myc in combination were incubated (5 h at 37°C) in medium with or without 10 nM CLC:CLF-1. Medium was recovered, and the cells were lysed in 1% Triton X-100 buffer as described above. Supernatants containing whole-cell extracts were analyzed for protein content using a protein assay (Bio-Rad), and the samples were subjected to immunoblot analysis with antibodies specific for sorLA, β-actin, and Myc tag.

For inhibition of lysosomal hydrolases, the cells were preincubated at 50 μg/ml leupeptin and pepstatin A (replaced every 6 h) for 24 h prior to continued incubation (37°C) in the presence of 10 nM CLC:CLF-1. The cells were subsequently either lysed as described above or fixed in PFA.

### Analysis of STAT3 phosphorylation.

Ba/F3 cells were starved for 3 h in cytokine-free medium prior to ligand stimulation. The cells were counted and seeded in 24-well plates with 1,200,000 cells per well. HEK293 and SH-SY5Y cells were seeded in 24-well plates and starved for 3 h prior to stimulation. Cells were incubated (15 min at 37°C) with or without CLC, CLF-1, CLC:CLF-1, CNTF, or CLC-sCNTFRα alone or with CLC:CLF-1 and sCNTFRα in combination at a concentration of 5 or 10 nM. Cells were lysed at 4°C in 1% Triton X-100 lysis buffer, as described above, supplemented with a phosphatase inhibitor cocktail (PhosSTOP; Roche). Supernatants containing whole-cell extracts were analyzed for protein content, and the samples were subjected to immunoblot analysis with antibodies specific for STAT3, pSTAT3, and β-actin.

### Western blotting and Coomassie staining.

Proteins were subjected to SDS-PAGE using either 4 to 16% gradient separation gels or NuPAGE 4 to 12% Bis-Tris protein gels (Invitrogen). For Western blotting, nitrocellulose membranes (Hybond-C; Amersham Biosciences, NJ) were blocked in 0.01 M Tris-HCl, 0.15 M NaCl, and 0.1% Tween 20, pH 7.6 (TBS-T) and 5% skimmed milk powder prior to incubation with antibodies in the same buffer. Membranes were washed in TBS-T containing 0.5% skimmed milk powder. Gels for Coomassie staining were incubated overnight in Coomassie staining solution containing 2.5 g of Coomassie brilliant blue (Merck) in 10% acidic acid, 40% ethanol, and 50% double-distilled H_2_O (ddH_2_O) and destained in 10% acidic acid, 40% ethanol, and 50% ddH_2_O afterwards.

## RESULTS

### CLC:CLF-1 targets sorLA.

**FIG 1:**
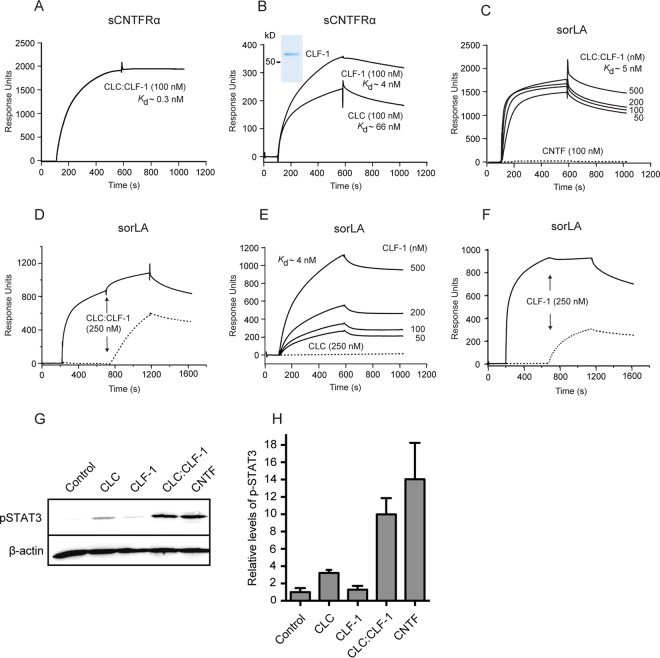
SPR analysis of the binding of CLC:CLF-1, CLC, and CLF-1 to immobilized sCNTFRα and to the immobilized luminal domain of sorLA, as indicated. (A) CLC:CLF-1 binding (at 100 nM) to sCNTFRα. (B) Binding of the individual subunits CLC and CLF-1 to sCNTFRα. Ligand concentrations and estimated *K_d_*s are indicated, and an SDS-PAGE analysis of the purified CLF-1 is shown in the inset (Coomassie stain). (C) Concentration dependence of CLC:CLF-1 binding to sorLA. The dotted line indicates CNTF binding. (D) Inhibition of CLC:CLF-1 binding to sorLA following preincubation of sorLA with sorLA-propeptide. Immobilized sorLA was first exposed to either unsupplemented binding buffer (dotted line) or to buffer containing sorLA propeptide (5 μM; solid line), and CLC:CLF-1 was subsequently added as indicated (arrows). (E) Concentration dependence (and *K_d_*) of CLF-1 binding to sorLA. The dotted line indicates binding of CLC (250 nM) to sorLA. (F) Inhibition of CLF-1 binding to sorLA preincubated with sorLA-propeptide. sorLA was subjected to unsupplemented buffer (dotted line) or to buffer containing a high concentration (5 μM) of sorLA propeptide (solid line) prior to addition of CLF-1 as indicated. (G and H) Induction of pSTAT3 in SH-SY5Y cells. The cells were starved for 4 h prior to stimulation (15 min) with 10 nM CLC, CLF-1, CLC:CLF-1, or CNTF (as a positive control) and subsequently lysed in the presence of phosphatase inhibitors. Induction of pSTAT3 was determined by reducing SDS-PAGE and Western blotting using anti-pSTAT3 antibodies. Panel G shows detected pSTAT3 and corresponding levels of β-actin of a single experiment. Results of four similar experiments (means ± SEM) are shown in panel H.

We have previously reported that the Vps10p-D receptor sortilin binds the two CNTFRα ligands CNTF and CLC:CLF-1 (20). Since sortilin and sorLA are members of the same receptor family and are known to share several ligands, we initially examined whether sorLA also exhibited affinity for the two ligands. We found that sorLA, unlike sortilin, did not bind to CNTF, whereas the heterodimeric CLC:CLF-1 bound to sCNTFRα as well as to the ectodomain of sorLA (Fig. 1A and C). Analysis by SPR demonstrated that binding to immobilized soluble sorLA, i.e., the luminal part of full-length sorLA, exhibited high affinity (*K_d_* of 5 ± 2.9 nM; *n* = 3), as determined by binding at different concentrations of CLC:CLF-1 (Fig. 1C). The binding was completely abolished in the presence of a surplus of sorLA's own propeptide, demonstrating that CLC:CLF-1 selectively targets the Vps10p-D β-propeller (Fig. 1D). Binding was also efficiently inhibited by receptor-associated protein (RAP) but almost unchanged by neurotensin (NT) (data not shown).

### sorLA selectively binds CLF-1, while CNTFRα binds both CLF-1 and CLC.

The affinity of CLC:CLF-1 for immobilized sCNTFRα was even higher, approximately 10-fold, than its affinity for sorLA, and the *K_d_* was estimated to be 0.3 ± 0.3 nM (mean ± standard deviation [SD]; *n* = 3) (Fig. 1A). To determine if one or possibly both elements of the heterodimer contribute to receptor binding, we first examined the activity of CLC. It appears (Fig. 1E) that CLC on its own was incapable of engaging sorLA and that its affinity for sCNTFRα was surprisingly modest (Fig. 1B), having a *K_d_* of 66 ± 39 nM (mean ± SD; *n* = 3), which is more than a 100-fold lower than that of the CLC:CLF-1 complex (Fig. 1A). Since CLC and the CLC:CLF-1 heterodimer were prepared from different expression systems (bacteria and insect cells, respectively), a direct comparison between the estimated *K_d_* values should be considered with some caution. However, similar results were obtained with CLC from several different batches, strongly indicating that CLF-1 could be a key player in the binding of CLC:CLF-1 to each of the two receptors. To test CLF-1 itself, we used a His-tagged CLF-1 construct purified from the medium of stably transfected CHO cells (Fig. 1B, inset). The subsequent SPR experiments showed that CLF-1 binds to sorLA with an affinity (*K_d_* = 4 ± 0.8 nM; *n* = 3) (Fig. 1E) very similar to that of the CLC:CLF-1 heterodimer (Fig. 1C) and that the interaction was inhibited by the sorLA propeptide (Fig. 1F) as well as by RAP (data not shown), whereas inhibition by NT was negligible (data not shown). It can be concluded that CLC:CLF-1 targets the Vps10p-D in sorLA by a single site residing in the CLF-1 subunit. Notably, CLF-1 also bound sCNTFRα (*K_d_* = 4 ± 1.3 nM; *n* = 3) (Fig. 1B) and, seemingly, with an affinity significantly higher than that of CLC. The latter finding suggests that the comparatively much higher affinity exhibited by the assembled CLC:CLF-1 heterodimer is owed to a bonus effect generated by two “minor” binding sites, one in each subunit.

We next examined CLF-1's contribution to CLC- and CNTFRα-mediated signaling. To that effect, CNTFRα-expressing SH-SY5Y cells were stimulated for 15 min with a 10 nM concentration of either CLC, CLF-1, or the CLC:CLF-1 complex, and the magnitude of induced signaling was subsequently measured by determining the resulting level of phosphorylated STAT3 (pSTAT3). The results (Fig. 1G and H) showed that whereas CLC:CLF-1 induced a strong signal, very similar to the response to 10 nM CNTF, stimulation with CLC resulted in only a modest increase in pSTAT3, and the pSTAT3 level upon CLF-1 stimulation did not differ from that of unstimulated controls.

Thus, CLF-1's role in signaling seems to reflect its contribution to the binding between CLC and CNTFRα. In other words, via complex formation with CLF-1, CLC gains a higher affinity for CNTFRα which enhances its accumulation on the cell membrane and formation of the tripartite signaling complex (CLC:CLF-1:CNTFRα) and, hence, its ability to promote engagement of gp130/LIFRβ for signaling.

### CNTFRα and sorLA connect via CLC:CLF-1.

**FIG 2:**
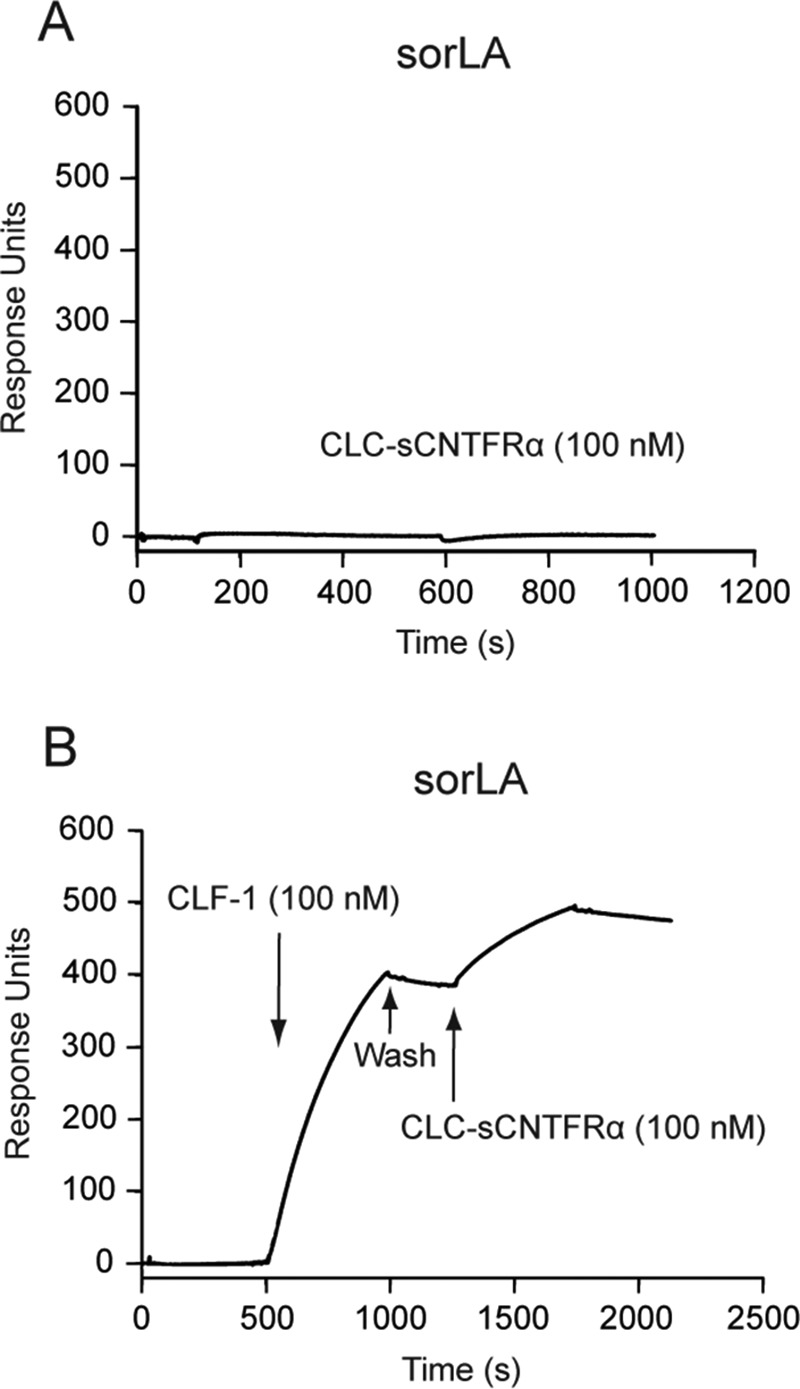
SPR analysis: CLF-1-mediated association between sorLA and the CLC-sCNTFRα fusion protein. (A) Binding of CLC-sCNTFRα (100 nM) to immobilized sorLA. (B) As indicated, immobilized sorLA was initially exposed to CLF-1 (100 nM). After binding, the chip was washed in unsupplemented buffer and finally subjected to fresh buffer containing 100 nM CLC-sCNTFRα. The subsequent increase in response units signifies binding of the fusion protein to preformed CLF-1:sorLA complex.

An additional SPR experiment was set up to determine if the CLC:CLF-1 heterodimer could in fact interact with sorLA and CNTFRα simultaneously. To that end, we first tested binding of a complex of sCNTFRα and CLC, in which the two were connected with a covalent linker peptide to prevent dissociation (CLC:sCNTFRα). As demonstrated by the results shown in Fig. 2A, this construct exhibited no binding activity toward immobilized sorLA, while a significant binding of the construct was seen after pretreatment of sorLA with CLF-1 (Fig. 2B). It can be concluded that CLC:CLF-1 may target both receptors at the same time and thereby constitute a connecting bridge between the two.

### sorLA mediates cellular uptake of CLF-1 and CLC:CLF-1.

**FIG 3:**
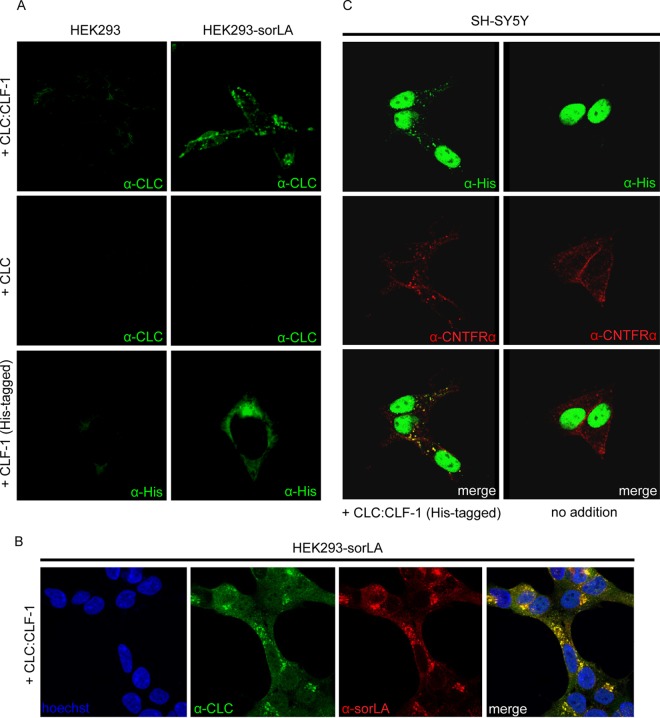
Cellular uptake of CLC, CLF-1, and CLC:CLF-1. (A) Untransfected and sorLA transfected HEK293 cells were incubated (37°C, 25 min) at 10 nM concentrations of the indicated ligands and then washed, fixed, and permeabilized. The cells were finally stained using mouse anti-His or rabbit anti-CLC as primary antibodies and Alexa Fluor 488-conjugated goat anti-mouse or anti-rabbit antibodies as secondary antibodies. (B) Colocalization of internalized CLC:CLF-1 and sorLA. sorLA transfected HEK293 cells were incubated with CLC:CLF-1 as described for panel A and subsequently stained with rabbit anti-CLC, mouse anti-sorLA, and matching secondary antibodies. (C) Colocalization of CLC:CLF-1 and endogenous CNTFRα (10 nM, 25 min). Following fixation the cells were stained with mouse anti-His, goat anti-CNTFRα, and appropriate secondary antibodies (Alexa Fluor 488-conjugated anti-mouse Ig and Alexa Fluor 568-conjugated anti-goat Ig).

Binding to full-length sorLA was studied in cells. Wild-type HEK293 cells and cells stably transfected with sorLA were incubated at 37°C in medium containing a 10 nM concentration of either CLC, His-tagged CLF-1, or CLC:CLF-1. After 25 min the cells were fixed, permeabilized, and stained with anti-CLC or anti-His tag antibodies to visualize internalized ligands. No staining was observed in untransfected cells, but in good agreement with the SPR results, CLF-1 and CLC:CLF-1, but not CLC, had been taken up (and internalized) by the sorLA transfectants (Fig. 3A). Double staining (for CLC:CLF-1 and sorLA) of cells from similar experiments demonstrated that the internalized ligand colocalized with sorLA (Fig. 3B). Similar results were obtained in sorLA transfected SH-SY5Y cells (data not shown), but in these cells, which express endogenous CNTFRα, internalized CLC:CLF-1 was furthermore seen to colocalize with CNTFRα (Fig. 3C).

**FIG 4:**
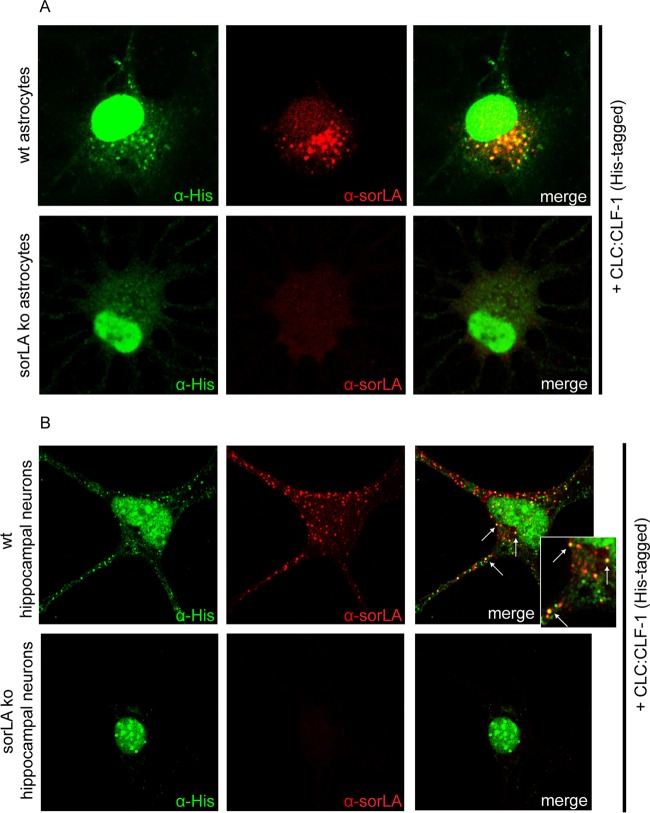
Uptake of CLC:CLF-1 is impaired in sorLA-deficient astrocytes and hippocampal neurons. (A) Astrocytes isolated from wt and from sorLA KO mice were incubated with His-tagged CLC:CLF-1 (40 nM at 37°C). The cells were then washed prior to fixation and finally permeabilized before staining with mouse anti-His and rabbit anti-sorLA antibodies. Alexa Fluor 488-conjugated donkey anti-mouse and Alexa Fluor 568-conjugated goat anti-rabbit antibodies were used as secondary antibodies. (B) Uptake of His-tagged CLC:CLF-1 in wt and sorLA KO mouse hippocampal neurons. The cells were incubated with CLC:CLF-1, fixed, and stained with anti-His and anti-sorLA antibodies as described above.

Uptake of CLC:CLF-1 was confirmed in astrocytes, which exhibit endogenous expression of sorLA but little or no CNTFRα as determined by Western blotting and lack of response to CLC:CLF-1 stimulation (data not shown). Cultured astrocytes isolated from wt and sorLA-deficient (KO) mice were incubated (25 min) in the absence or presence of 40 nM His-tagged CLC:CLF-1 prior to staining with anti-His and anti-sorLA antibodies. As demonstrated in Fig. 4A, sorLA-deficient cells showed little or no uptake of CLC:CLF-1, whereas wt astrocytes presented a significant vesicular uptake, which to a large extent colocalized with sorLA. Thus, as determined by manual counting, wt astrocytes contained 11.44 ± 4.02 (mean ± SD; *n* = 16) CLC:CLF-1-positive vesicles per cell in contrast to the 1.25 ± 1.29 (*n* = 16) positive vesicles per cell found in sorLA-deficient astrocytes. A similar accumulation of ligand colocalizing with sorLA was observed in isolated wt hippocampal (mouse) neurons expressing sorLA but not in sorLA-deficient hippocampal neurons (Fig. 4B).

### sorLA mediates internalization of CNTFRα via binding to CLC:CLF-1.

**FIG 5:**
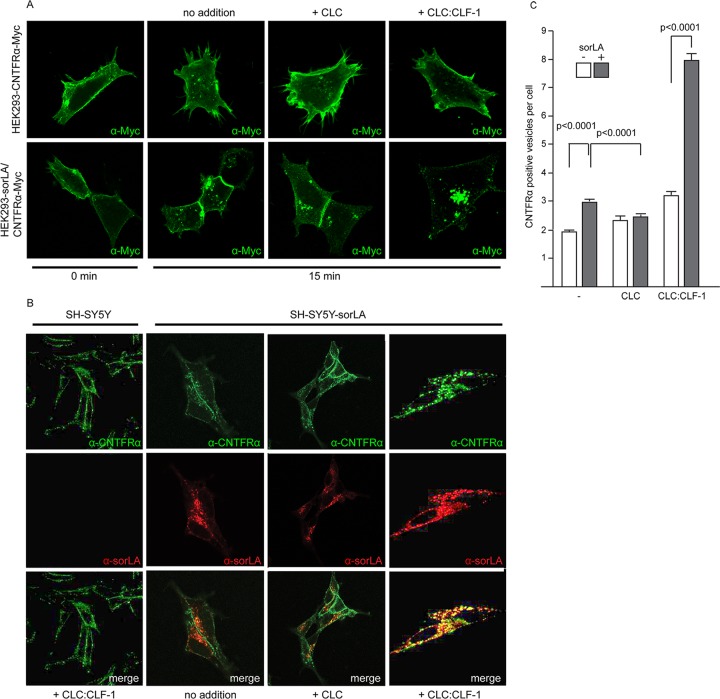
sorLA-mediated internalization of CNTFRα. (A) HEK293 cells transfected with Myc-tagged CNTFRα or doubly transfected with CNTFRα-Myc and sorLA were incubated (37°C, 10 min) with mouse anti-Myc, washed, and then incubated with the given ligands (at 10 nM). At the given times the cells were fixed, permeabilized, and stained with Alexa Fluor 488-conjugated goat anti-mouse Ig. (B) Internalization and colocalization of CNTFRα (endogenous) and sorLA in SH-SY5Y transfectants. Wild-type cells and sorLA transfectants were incubated (37°C for 25 min) with the given ligands (at 10 nM) and then fixed, washed, permeabilized, and finally stained by goat anti-CNTFRα and mouse anti-sorLA. Alexa Fluor 488-conjugated donkey anti-goat and Alexa Fluor 568-conjugated donkey anti-mouse antibodies were used as secondary antibodies. (C) Number of CNTFRα-containing vesicles in wt and sorLA transfected SH-SY5Y cells. The cells were incubated with ligands and stained as described for panel B prior to automated counting of vesicles with a positive stain for CNTFRα. Each column represents results obtained in 346 to 2,047 cells (containing between 800 and 9,444 positive vesicles) and shows mean values ± SEMs.

The SPR data (Fig. 2B) and the findings in SH-SY5Y cells suggested that the CLC:CLF-1 heterodimer might serve as a molecular link between sorLA and CNTFRα, and this prompted us to examine if sorLA, via binding to CLC:CLF-1, also conveyed uptake of CNTFRα. HEK293 cells expressing CNTFRα-Myc alone or both receptors in combination were fixed and stained with an anti-Myc antibody before and after incubation (37°C) in unsupplemented medium or in medium supplemented with 10 nM CLC or CLC:CLF-1. It appears from the subsequent fluorescence microscopy (Fig. 5A) that in the absence of sorLA nearly all CNTF receptors were localized to the surface membrane both before and after incubation with ligands. In contrast, distinct intracellular staining, signifying internalized CNTFRα, was seen in the double transfectants. Staining was particularly strong in the cells treated with CLC:CLF-1, but minor staining was also seen upon stimulation with CLC and, surprisingly, in cells not subjected to either of the two ligands.

Similar results were obtained by automated counting of CNTFRα-positive vesicles in sorLA transfected and in untransfected SH-SY5Y cells, which constitutively express CNTFRα (Fig. 5B and C).

As shown in Fig. 5B, internalization of CNTFRα in the absence of ligands was more pronounced in sorLA transfected than in wt SH-SY5Y cells, and this difference was strongly enhanced in the presence of CLC:CLF-1. However, in cells subjected to medium containing CLC, sorLA's promoting effect on internalization of CNTFRα appeared to be eliminated or, compared to internalization in sorLA transfectants not exposed to ligands, even reversed (Fig. 5C).

### sorLA and CNTFRα may interact directly.

**FIG 6:**
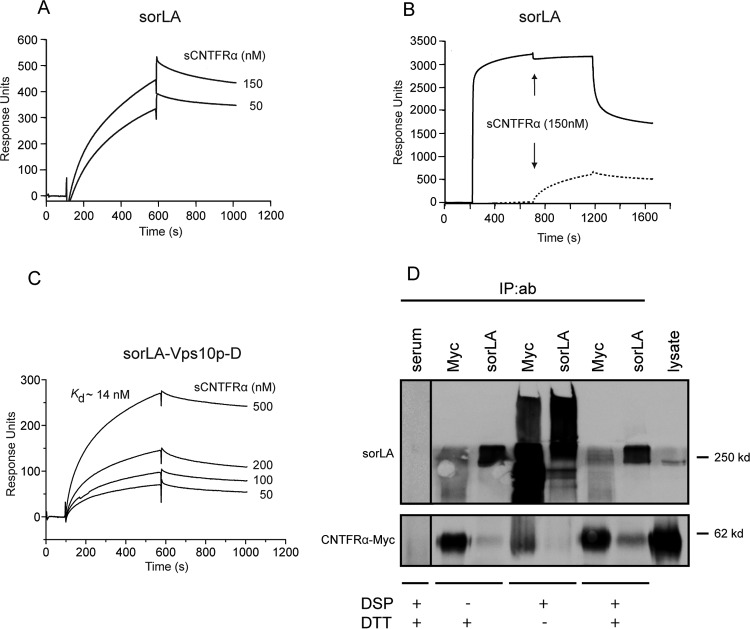
Interaction between the luminal domains of CNTFRα and sorLA. (A) Concentration-dependent binding of sCNTFRα to the immobilized luminal (entire extracellular) part of sorLA. (B) RAP-mediated inhibition of the interaction between sCNTFRα and sorLA. Immobilized sorLA was exposed to unsupplemented buffer (dotted line) or to buffer supplemented with 5 μM RAP (solid line) prior to the addition of sCNTFRα (arrows). (C) Concentration dependence of sCNTFRα binding to the immobilized Vps10p-D of sorLA. (D) Cross-linking of full-length receptors in cells. HEK293 double transfectants expressing CNTFRα-Myc and sorLA were incubated with the chemical cross-linker DSP (45 min, 2 nM), and receptor proteins were precipitated from lysates using anti-Myc and anti-sorLA antibodies as indicated. Reduced (+DTT) and unreduced samples of precipitate were analyzed by SDS-PAGE. IP, immunoprecipitation; ab, antibody.

Whereas the former result confirmed that CLC:CLF-1 may bind both receptors simultaneously and thus promote sorLA-mediated endocytosis of CNTFRα, the latter seemed to suggest that the mere presence of sorLA was enough to facilitate a significant degree of endocytosis and that this effect was blocked by CLC, which binds CNTFRα but not sorLA (Fig. 1E). To clarify this, we first performed SPR analysis to determine if the ectodomains of the two receptors interacted. Soluble CNTFRα bound to immobilized sorLA and competed with RAP for binding (Fig. 6A and B). RAP interacts with both the complement-type repeats and the Vps10p-D of sorLA, but as further analysis demonstrated specific binding of sCNTFRα to the purified Vps10p-D (Fig. 6C), this domain must harbor the binding site. Since sCNTFRα in complex with CLC did not associated with sorLA (Fig. 2A), it can be deduced that the two receptors can interact directly in the absence of ligands or interact indirectly (by proxy) in the presence of CLC:CLF-1 and that CLC, when complexed to CNTFRα, inhibits/blocks the interaction.

### CNTFRα forms a complex with full-length sorLA in cells.

Direct interaction between full-length receptors in cells was next pursued by cross-linking. HEK293 cells expressing either CNTFRα-Myc or sorLA or both in combination were incubated with the chemical cross-linker DSP at room temperature. After 15 min, the reaction was stopped by addition of Tris. The cells were then washed and dissolved in lysis buffer, and following removal of debris by centrifugation, supernatants were subjected to immunoprecipitation using anti-Myc and anti-sorLA Ig and finally analyzed by native and reducing SDS-PAGE (Fig. 6D). The analysis showed that only one band, representing the expressed receptor, could be precipitated from single transfectants and only by the relevant receptor-specific antibody (data not shown). In double transfectants, however, both antibodies precipitated high-molecular-weight adducts which, in the presence of dithiothreitol (DTT) (cleaving the cross-linker), resolved in two bands representing CNTFRα and sorLA. In view of this result, the above SPR analysis, and the findings using fluorescence microscopy (Fig. 5C), we conclude that sorLA and the CNTFRα interact on cell membranes.

### Following binding of CLC:CLF-1, CNTFRα is downregulated by sorLA.

**FIG 7:**
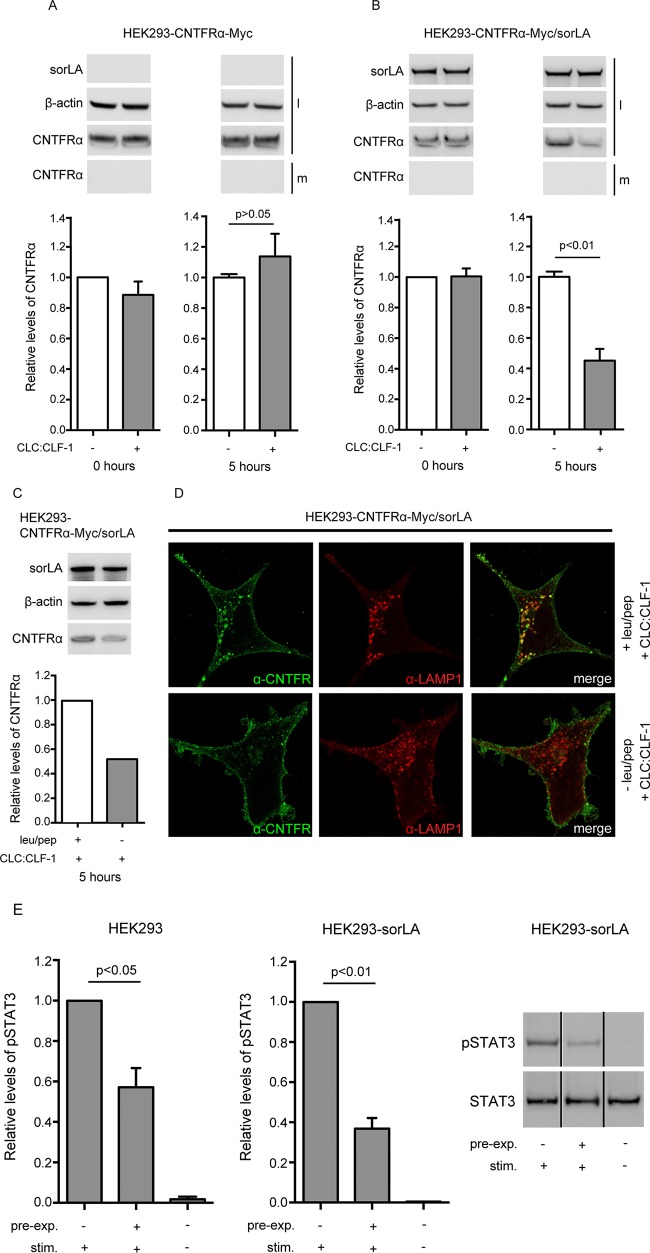
sorLA mediates CLC:CLF-1-dependent downregulation of CNTFRα. (A and B) HEK293 single transfectants expressing CNTFRα-Myc (A) and double transfectants expressing both CNTFRα-Myc and sorLA (B) were incubated in the absence (−) or presence (+) of 10 nM CLC:CLF-1. Incubation was stopped (time zero or after 5 h), and the content of CNTFRα-Myc found in the medium (m) and in cell lysates (l) was detected by Western blotting and quantified by densitometry of specific bands. Upper panels show Western blot results from a typical experiment. Lower panels show the detected levels of CNTFRα-Myc found in cell lysates. The levels are shown relative to the CNTFRα-Myc level in single transfectants at time zero. Data represent means ± SEMs of three experiments. (C) Western blotting and quantitation of CNTFRα in cells subjected or not to lysosomal inhibitors prior to incubation (5 h) with CLC:CLF-1. (D) Immunofluorescence showing accumulation of CNTFRα in LAMP-1-positive vesicles of cells treated with lysosomal inhibitors. (E) HEK293 cells were preincubated in the absence or presence of 10 nM CLC:CLF-1 for 5 h (pre-exp.), washed, starved in unsupplemented medium for 1.5 h, and finally restimulated (stim.) with 5 nM CLC:CLF-1, as indicated, for 15 min. The columns show the relative levels of pSTAT3 in wt HEK293 cells and in sorLA transfectants. Data represent means ± SEMs (*n* = 3) relative to the pSTAT3 level in cells preincubated in the absence of CLC:CLF-1 but restimulated with CLC:CLF-1. The right panel shows a Western blot of the response (pSTAT3) obtained in an experiment with cells overexpressing sorLA.

To determine if sorLA-mediated internalization of the CLC:CLF-1:CNTFRα complex was accompanied by downregulation (degradation) of CNTFRα, we initially tried to detect downregulation using biolabeled receptors. However, as labeling was poor due to a very modest synthesis of CNTFRα at steady state, we instead decided to measure the total pool of CNTFRα in HEK293 transfectants before and after incubation with CLC:CLF-1. Single transfectants expressing CNTFRα-Myc and double transfectants expressing both CNTFRα-Myc and sorLA were incubated at 10 nM CLC:CLF-1 or in unsupplemented medium. After 0 and 5 h of incubation, the cells were lysed, and their content of CNTFRα was quantified by Western blotting. The results are shown in Fig. 7, and it appears (Fig. 7B) that in the double transfectants the cellular pool of CNTFRα was reduced by more than 50% upon 5 h of exposure to CLC:CLF-1. In contrast, the bulk of CNTFRα in the single transfectants (subjected to CLC:CLF-1 or not) remained virtually unchanged (showing a slight but insignificant increase) upon incubation with CLC:CLF-1 (Fig. 7A). Furthermore, downregulation of CNTFRα was almost absent in cells treated with the lysosomal inhibitors leupeptin and pepstatin A (Fig. 7C) and was instead seen to accumulate in LAMP-1-positive vesicles (Fig. 7D). Thus, our data demonstrate that CNTFRα, following endocytosis in complex with CLC:CLF-1 and sorLA, is directed toward degradation in lysosomes rather than recycled to the surface membrane.

In accordance with the above (downregulation of CNTFRα), we found that sorLA lowered the response to renewed stimulation in cells that had been preexposed to CLC:CLF-1. This was seen in wt HEK293 cells, which have modest endogenous expression levels of both CNTFRα and sorLA. The cells were first preincubated in the absence or presence of CLC:CLF-1 (5 h, 10 nM), then washed and incubated in unsupplemented medium (1.5 h), and finally restimulated with CLC:CLF-1 (5 nM for 15 min). Signal induction, in terms of pSTAT3, was subsequently measured by Western blotting of cell lysates. The results showed that cells which had been preincubated with CLC:CLF-1 presented a much weaker response to restimulation than control cells preincubated in blank medium. Thus, in three experiments, the level of pSTAT3 in preexposed cells was only 57.17% ± 9.51% (mean ± standard error of the mean [SEM]) of that in control cells (Fig. 7E, left panel), and, notably, the pSTAT3 level in transfected HEK293 cells overexpressing sorLA (and tested in parallel with the wt cells) was even lower than that in wt cells (although not significantly lower; *P* = 0.13), constituting as little as 36.83% ± 5.35% (mean ± SEM) of the level in controls (Fig. 7E, right panels).

### sorLA may facilitate signaling in cells not expressing CNTFRα.

**FIG 8:**
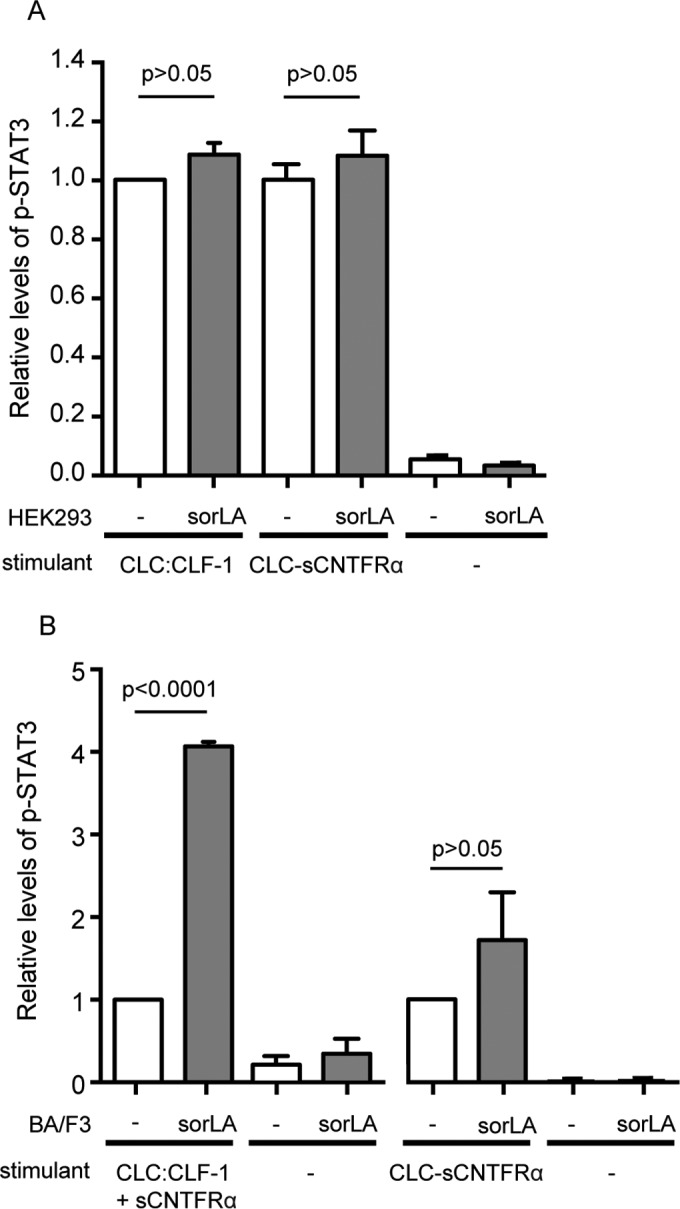
sorLA's influence on CLC:CLF-1 signaling in cells with or without endogenous expression of CNTFRα. (A) CNTFRα-expressing HEK293 cells were stimulated (15 min) with 10 nM CLC:CLF-1 or with the fusion protein CLC-sCNTFRα as indicated. The columns show the response, in terms of pSTAT3, in wt HEK293 cells (−) and in sorLA transfectants. Data represent means ± SEMs (*n* = 3) relative to the pSTAT3 level in wt cells stimulated with CLC:CLF-1. (B) Levels of pSTAT3 in CNTFRα-deficient (but gp130/LIFRβ-positive) Ba/F3 cells upon stimulation with a combination of 10 nM CLC:CLF-1 and sCNTFRα or with the CLC-sCNTFRα fusion protein. Results in sorLA transfectants (means ± SEMs, *n* = 3) are shown relative to results obtained in wt cells (−).

To determine if sorLA impacts cellular signaling via its ability to interact with CLC:CLF-1 bound to CNTFRα, we first examined STAT3 phosphorylation in HEK293 cells with endogenous expression of CNTFRα. The cells were untransfected or transfected with sorLA and were stimulated for 15 min with either 5 nM CLC:CLF-1 or a 5 nM concentration of the fusion protein CLC-sCNTFRα. The pSTAT3 level was measured by Western blotting, and the results are shown (Fig. 8A) relative to the level obtained in untransfected HEK293 cells responding to CLC:CLF-1. As can be seen, expression of sorLA did not significantly alter the response. A similar experiment was next performed in CNTFRα-deficient Ba/F3 cells (expressing gp130/LIFRβ), but in this case stimulation with CLC:CLF-1 was performed in the presence of sCNTFRα (Fig. 8B). Again the responses to stimulation with the fusion protein, which does not bind sorLA, did not differ significantly between untransfected cells and sorLA transfectants. However, in cells subjected to CLC:CLF-1 and sCNTFRα, the resulting level of pSTAT3 was about 4-fold higher in the sorLA transfectants than in the untransfected Ba/F3 cells. Taken together, the two experiments strongly indicate that sorLA may serve to bind and concentrate circulating tripartite complexes of CLC:CLF-1 and sCNTFRα on the cell membrane and thereby promote their interaction with gp130/LIFRβ and signaling. This effect appears to be insignificant on cells that express CNTFRα but may have a significant effect on cells without endogenous expression of CNTFRα.

## DISCUSSION

Like CNTF, the heterodimeric cytokine CLC:CLF-1 primarily targets the GPI-anchored receptor CNTFRα. The resulting tripartite complex subsequently recruits the gp130/LIFRβ signal-transducing receptors for signaling via the JAK/STAT3 pathway (36–38).

CLC and CLF-1 are (co)expressed in a large number of cell types both inside and outside the nervous system (39), and, like CNTF, the CLC:CLF-1 heterodimer as well as the individual CLC subunit promotes growth and survival of CNTFRα-expressing neuronal cells (5, 11, 40). In the presence of CNTFRα (membrane anchored or soluble), CLC is sufficient to induce signaling, whereas CLF-1, as exemplified in the results shown in Fig. 1G and H, has no independent capacity for signal induction (41).

Unlike CNTF, neither CNTFRα nor CLC:CLF-1 is physiologically redundant, and in contrast to CNTF-deficient mice, which appear normal and healthy (42), CLC-, CLF-1-, or CNTFRα-deficient mice suffer from a decreased number of neurons, notably in the nucleus facialis and in motor neurons, and die shortly after birth due to a failure to suckle (7, 9–11).

Similar observations have been made in humans. Thus, apart from an insignificant (and seemingly harmless) decrease in motor neurons in old age, lack of CNTF appears to be innocuous (43), while compound mutations causing insufficient expression of CLC and/or CLF-1 result in a series of defects manifested in the form of human CISS/Sohar-Crisponi syndrome (8, 12–16, 44). Some of the cardinal symptoms in CISS, e.g., the risk of early death (originating from motor neuron defects and the resulting inability to suckle) is very similar to findings in the KO animals.

As demonstrated by results in both humans and mouse, each of the three subunits in the signaling tripartite complex CLC:CLF-1:CNTFRα is indispensable *in vivo*; i.e., a functional deficiency in either one of the three subunits is enough to cause neonatal death in mice and full-blown CISS in humans. With regard to CLC and CNTFRα this is not surprising, as both are needed for interaction with gp130/LIFRβ and the subsequent signaling events. However, CLF-1, alone or in combination with CNTFRα, has no direct impact on signaling, and so far its only know function is to facilitate transport and secretion of CLC. As previously described, CLC is expressed in the biosynthetic pathway but seems to depend on coexpression and complex formation with CLF-1 (or possibly CNTFRα) for cellular release (4, 39, 45). The clinical manifestations of CLF-1 deficiency have therefore been ascribed to the resulting decrease in CLC secretion. However, a recent study (41) has provided compelling evidence for a more complex function. Thus, it appears that some CLF-1 mutations may cause disease without seriously affecting the cellular secretion of CLC, which implies that CLF-1 is more than just a facilitator of CLC secretion.

### CLF-1 is as a key player in CLC:CNTFRα signaling and turnover.

In this study, we show that, apart from facilitating the cellular release of CLC, CLF-1 has at least two other functions, i.e., one that has to do with CLC:CNTFRα complex formation and signaling and one that implicates a new receptor with a significant impact on the turnover of CLF-1 itself as well as of its binding partners CLC and CNTFRα.

### CLF-1 sustains CLC binding to CNTFRα and thereby promotes CLC signaling.

Despite the fact that CLC, unlike CLF-1, is a prerequisite for the recruitment of gp130/LIFRβ and signaling (41), the CLC:CLF-1 complex is a much stronger inducer of STAT3 phosphorylation than the individual CLC subunit. The reason for this is revealed by the present binding experiments which show that, compared to CLC:CLF-1, CLC binds poorly to CNTFRα. Thus, CLC:CLF-1 binds with a *K_d_* which is about a 100-fold lower than that of CLC, and in effect CLC seems to gain a much higher affinity via complex formation with CLF-1. Since CLC and CLF-1 (as determined by gel filtration) tend to form polymers and even aggregates and as CLC:CLF-1 may dissociate into subunits, the reported affinities should, of course, be considered with some caution; the observed gain of affinity might, for instance, originate from a more stable and optimal conformation of CLC upon binding to CLF-1. Another factor that might explain or contribute to the shift in the *K_d_* is, as previously mentioned, the different expression systems used to generate the two reagents. Thus, the possibility that CLC generated in bacteria has a poor fold and a low binding ability compared to those of CLC and CLC:CLF-1 purified from cell cultures (or lysates) cannot be excluded (46). Yet our present findings suggest an alternative and perhaps more likely explanation. As demonstrated by the SPR analysis, CLF-1 comprises independent sites for binding of both CLC and CNTFRα. Also, it exhibits a similar or perhaps even higher affinity than CLC for CNTFRα, and, notably, it can engage both targets at the same time. In other words, whereas CLC comprises a single receptor binding site and has a relatively modest affinity, the CLC:CLF-1 complex contains two sites which in combination create a bonus effect and a much higher affinity for CNTFRα.

It can be concluded that in addition to its established function as a chaperone that facilitates transport and secretion of CLC in the biosynthetic pathway, CLF-1 may also play a decisive role in extracellular events involving the CNTFRα binding and signaling capacity of CLC. Previous findings have shown that a CLC-sCNTFRα fusion protein in which a linker peptide connects the two subunits provides a much stronger cellular stimulant than a 1:1 mixture of the two unlinked subunits (47). With that in mind, CLF-1 can be regarded as a sort of *in vivo* linker protein inasmuch as it binds both CLC and CNTFRα and thereby promotes their mutual interaction, their subsequent mobilization of the gp130/LIFRβ heterodimer, and ultimately their signal induction in responding cells. It has so far been generally accepted that the CLC subunit alone accounts for the binding of CLC:CLF-1 to the CNTFRα; the present findings do not exclude this possibility, but they do substantiate a likely and alternative mode of binding which includes the participation of the CLF-1 subunit.

### Functional aspects of CLF-1's interaction with the Vps10p-D receptor sorLA.

Since CLF-1 and CLC are not always coexpressed, it could be speculated that CLF-1 have CLC-independent functions and perhaps target alternative ligands and receptors. Previous findings suggest that the p28 subunit of IL-27 may represent such an alternative ligand (19), but, apart from CNTFRα (as shown in this study) potential CLF-1-specific receptors have not been reported until now. CLF-1 binds to sorLA's Vps10p-D with an affinity similar to or higher than its affinity for CNTFRα. Notably, sorLA does not interact with CLC but binds the CLC:CLF-1 heterodimer just as well as CLF-1, signifying that CLF-1 comprises at least three binding sites: one for CLC, one for CNTFRα, and one for sorLA. The sites are functionally independent, and complex formation with one partner does not prevent or compromise simultaneous interaction with either of the two others. Ultimately, CLF-1 can bind all its targets at the same time and in this way link both CLC and CNTFRα to sorLA.

sorLA is expressed both on the cell membrane and in intracellular compartments, and its cytoplasmic tail contains functional motifs for adaptors (e.g., GGAs and AP-1 and -2) involved in endocytosis as well as Golgi compartment-endosome trafficking (27, 29, 48). We have previously demonstrated that sorLA may sort certain newly synthesized proteins from the Golgi compartment to lysosomes and thereby regulate their cellular release (30). This is not the case with CLF-1. CLF-1 binds to the Vps10p-D of sorLA, and this domain contains a propeptide, which prevents domain-specific ligand binding in the Golgi compartment (26). To engage ligands targeting the Vps10p-D, sorLA must be subjected to propeptide cleavage, and as this event takes place after passage through the Golgi compartment, secretion of CLF-1 and/or CLC:CLF-1 is not affected by the presence of sorLA. However, sorLA mediates highly efficient uptake and endocytosis of CLF-1 and CLC:CLF-1 and appears to be a major player in the turnover of both. In accordance, we found rapid and significant cellular internalization of both ligands in sorLA transfected cell lines and in wt astrocytes and hippocampal neurons but nearly no internalized ligand in sorLA knockouts. Evidently, sorLA is a potent clearance receptor for removal of CLF-1 and the CLC:CLF-1 complex, but via its affinity for CLF-1 it also has a considerable impact on the turnover and expression of the CNTFRα.

### sorLA conveys CLF-1-dependent downregulation of CNTFRα.

Our cross-linking data and SPR analysis demonstrate direct binding between sorLA and CNTFRα, and our findings in cells suggest that this mechanism may account for a minor fraction of internalized CNTFRα. The direct interaction is abrogated when CNTFRα is in complex with a ligand (CLC), but when complexed with CLC:CLF-1, the CLF-1 subunit takes over and reestablishes the binding to sorLA, which in turn promotes endocytosis of the entire CLC:CLF-1:CNTFRα complex. As a result, sorLA-expressing cells subjected to prolonged stimulation with CLC:CLF-1 exhibit substantial downregulation of CNTFRα due to lysosomal degradation of internalized receptors and a low level of CNTFRα-synthesis.

**FIG 9:**
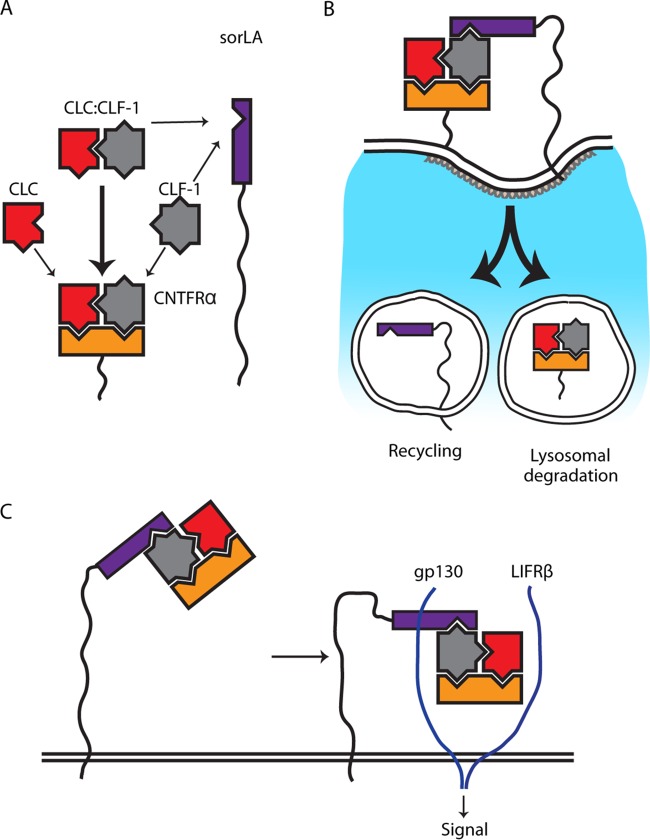
Model of the interplay between CLC, CLF-1, CNTFRα, and sorLA. (A) The cytokine subunits CLC and CLF-1 each contain a single site for CNTFRα binding and bind with moderate affinity (thin arrows). The assembled heterodimer CLC:CLF-1 contains two binding sites (one in each subunit), which together generate a bonus effect and a high affinity for CNTFRα (thick arrow). CLF-1 further harbors an independent site for binding to sorLA and binds sorLA alone or in complex with other subunits. (B) sorLA conveys endocytosis of the tripartite complex CLC:CLF-1:CNTFRα via binding to CLF-1. Following internalization, CNTFRα and CLC:CLF-1 are sorted to lysosomal degradation while sorLA is recycled. (C) sorLA may enhance signaling, notably in CNTFRα-deficient cells, by binding and concentrating complexes of CLC:CLF-1 and sCNTFRα on the cell membrane.

Following preexposure to CLC:CLF-1 (and sorLA-mediated downregulation of CNTFRα), the cellular response to renewed CLC:CLF-1 stimulation was significantly reduced in wt cells with endogenous expression of CNTFRα and sorLA and even more so in transfectants overexpressing sorLA. Thus, sorLA may have a profound effect on the cellular response under conditions of constant exposure to ligand (CLC:CLF-1), but apart from that, sorLA did not directly influence signaling; i.e., it neither enhanced nor hampered interaction with the gp130/LIFRβ heterodimer in CNTFRα-expressing cells. However, in cells expressing gp130/LIFRβ but not CNTFRα, expression of sorLA enhanced signaling (pSTAT3) in response to sCNTFRα in complex with CLC:CLF-1 but not to the CLC-sCNTFRα fusion protein complex, which does not bind sorLA. These results suggest that in cells without membrane-expressed CNTFRα, sorLA may bind and concentrate the tripartite soluble complex (CLC:CLF-1:sCNTFRα) on the cell membrane and in this way promote interaction with gp130/LIFRβ and thereby signaling. A previous study based on molecular modeling reported an overlap between CLC's binding sites for CLF-1 and LIFRβ, suggesting that CLF-1 has to be dissociated/removed to allow signaling, i.e., interaction between CLC and LIFRβ. However, we have recently generated a CLC–linker peptide–CLF-1 fusion protein (unpublished data) which binds both CNTFRα and sorLA and which is fully capable of signal induction. Since this indicates that CLF-1 may well be part of the signaling complex, we have included it in our proposed model (Fig. 9C).

### Similar functions in different settings?

Our study has revealed new functional aspects of CLF-1 and sorLA in the regulation of CLC:CNTFRα signaling and turnover, but it also brings a new perspective to previous observations concerning (i) the sorLA-related receptor sortilin and (ii) the IL-27 subunit p28. Thus, sortilin exhibits high affinity for CLC:CLF-1 (20), and CLF-1 may interact with p28 to form a cytokine complex which, in the presence of IL-6Rα, activates NK and T cells (19). Whether the findings reflect that sortilin plays a CLF-1-dependent role similar to that of sorLA and/or that CLF-1 implicates sorLA (and possibly sortilin) in p28 functions are among the obvious subjects for future research.
